# Silverleaf (*Chondrostereum purpureum*) Effects on Japanese Plum (*Prunus salicina*)

**DOI:** 10.3390/plants10122777

**Published:** 2021-12-16

**Authors:** Daina Grinbergs, Javier Chilian, Carla Hahn, Marisol Reyes, Mariana Isla, Andrés France, Jorunn Børve

**Affiliations:** 1 Laboratorio de Fitopatología de Frutales INIA Quilamapu, Instituto de Investigaciones Agropecuarias, INIA, Av. Vicente Méndez 515, Chillán 3780000, Chile; carlahahn@gmail.com (C.H.); mreyes@inia.cl (M.R.); marisla@udec.cl (M.I.); afrance@inia.cl (A.F.); 2 Norwegian Institute of Bioeconomy Research, NIBIO, P.O. Box 115, 1431 Ås, Norway; Jorunn.Borve@nibio.no

**Keywords:** Silverleaf disease, *Chondrostereum purpureum*, *Prunus salicina*, Japanese plum

## Abstract

Silverleaf is an important fungal trunk disease of fruit crops, such as Japanese plum (*Prunus salicina*). It is known that infection by *Chondrostereum purpureum* results in discolored wood, “silvered” foliage, and tree decline. However, effects on fruit yield and quality have not been assessed. Therefore, the objectives of this study were to determine *C. purpureum* pathogenicity on *P. salicina* and the effects on physiology, fruit yield, and quality, in Chile, in 2019 and 2020. Wood samples from affected plum trees were collected in the Chilean plum productive area. Fungi were isolated by plating wood sections from the necrosis margin on culture media. Morphological and molecular characteristics of the isolates corresponded to *C. purpureum* (98%). Representative isolates were inoculated from healthy plum plants and after 65-d incubation, wood necrotic lesions and silver leaves were visible. Fungi were reisolated, fulfilling Koch’s postulates. To determine Silverleaf effects, xylem water potential and fruit yield and quality were measured in healthy and Silverleaf-diseased plum trees ‘Angeleno’. Water potential was altered in diseased trees, and fruit yield was reduced by 51% (2019) and by 41% (2020) compared to fruit from healthy trees. Moreover, cover-colour, equatorial-diameter, and weight were reduced, and fruit were softer, failing to meet the criteria to be properly commercialized and exported to demanding markets.

## 1. Introduction

The Japanese plum (*P. salicina* L.) is a deciduous stone fruit tree native to China. It is grown globally, and Chile cultivates 4520 ha, mainly ‘Angeleno’, ‘Black-Amber’, and ‘Friar’ cultivars. Chilean production volume is ~400,000 t (2019–2020), and Chile is the major fresh plum exporter worldwide [1].

Fungal trunk diseases have increased in recent years, in fruit, ornamental, forest, and woody species, worldwide [2]. Some of the reasons of this increase in trunk diseases in fruit crops are changes in climate, the incorporation of intensive plant production management measures such as high density plantations, the use of dwarfing rootstocks, and severe pruning, in order to accelerate plant and fruit production, which stress the plants and alter their behaviour [2,3,4]. Fungal trunk diseases are one of the main pathological problems for *Prunus*, which are affected by several fungal taxonomic groups [5]. *Prunus salicina* has been reported as a host of different wood fungal pathogens, such as the ones from the genera *Armillaria* [6], *Botryosphaeria* [7], *Diplodia* [8], *Calosphaeria*, *Jattaea* [9], *Lasiodiplodia* [10], *Neofusicoccum* [11], *Phaeoacremonium*, *Tonignia* [12], and *Chondrostereum* [13].

The basidiomycete *C. purpureum* (Pers.) Pouzar is an important wood pathogen that causes Silverleaf disease. While this fungal species has attracted considerable interest in several countries as a biological agent for woody weed control in coniferous forest plantations [13,14,15,16,17], it can cause severe and destructive disease in woody plants, including ornamental, wild, forest, and fruit crop species in some areas of the world [4,17,18,19,20,21,22,23]. More than 230 species are recorded as hosts of *C. purpureum* [15,18,19,24,25], i.e., the Rosaceae family [3,4,18,19,26,27,28] and genus *Prunus*, one of the most susceptible to this pathogen [18], such as *P. armeniaca* [29], *P. avium* [30], *P. persica* 49 [31], *P. serotina* [19], *P. domestica* [32], and *P. salicina* [13].

This fungus reproduces through basidiospores, which are airborne disseminated from basidiocarps, developing in dying trunks and branches of infected trees, to fresh wood wounds [33]. Mycelia grow through the xylem tissue, producing central wood discolouration [34] and subsequently occluding the vessels. In addition, the fungus produces a specific endopolygalacturonase (endoPG) enzyme, which moves to the foliage, inducing silver-greyish colour symptoms on leaves. This silver colour is usually visible several seasons after the infection [27,35]. Subsequently, the fungus kills the plant and develops purple resupinated carpophores that release spores under high humidity and mild temperatures [33].

Silverleaf is an important disease in countries such as New Zealand, the United States (Washington and Oregon) [32], Chile [3], Australia [36], France [18], Poland [37], Latvia [38], Norway [39], and other European countries [32]. It can produce severe losses in orchards, and it is the main cause of mortality in peach and nectarine in New Zealand, with losses of 8% per year [40]. In Chile, it has caused disease in different fruit crop species, such as almond, apples, blueberries, cherry, Chilean guava, nectarine, quince, pear tree, and plum [3,4,23], showing silver leaves and brown central discoloration in branches and the main trunks. In blueberry, the effects of Silverleaf on plants and fruit were measured, with water potential and stomatal conductance the most affected physiological parameters, as well as a yield reduction of 40% and problems in fruit quality, such as reduction of maturation, colour, and weight [4]. Nevertheless, there is a lack of information about the effects of Silverleaf in other fruit crops.

New wood pathogens are being reported every day, and in a wider range of hosts and geographical locations [5,9,12,17,28,41,42]. However, little is known about their impact on fruit yield and quality. The most exhaustive research has been performed on grapevines, revealing that wood diseases are the main biotic factor limiting vineyard productivity and longevity, causing major economic losses [43].

Similar to that for other other trunk diseases, the information about Silverleaf effects on fruit crops and in Japanese plum is scarce. Therefore, the objectives of the present study were to investigate the etiology of the fungal pathogen causing foliar silvering in *P. salicina* in Chile, to determine its pathogenicity in plum, and to assess the effects of *C. purpureum* on plum physiology, fruit yield, and quality.

## 2. Results

### 2.1. Chondrostereum purpureum Isolates

**Figure 1:**
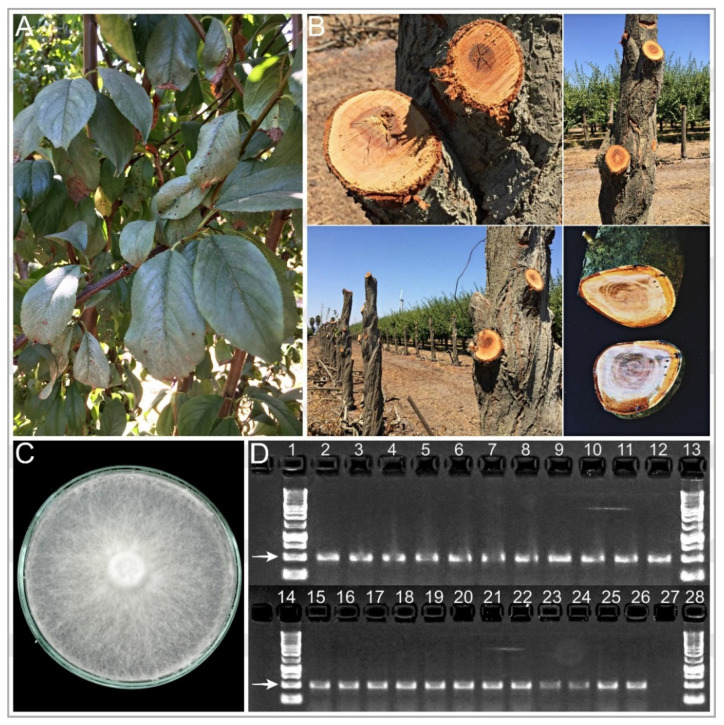
Silverleaf disease caused by *Chondrostereum purpureum* on Japanese plum in Chile; (**A**) foliar symptoms and (**B**) wood discoloration symptoms on plum trees ‘Angeleno’, (**C**) isolate HMCi7 Genbank: MW938164, and (**D**) Polimerase Chain Reaction (PCR) products using APN1 species-specific primers for 22 *C. purpureum* isolates (Lanes 2 to 11 and 15 to 26). Lane 12: positive control isolate RGM 122 GenBank: MK22253.1. Lane 27: negative control. Lanes 1, 13, 14, and 28: molecular weight standards. The white arrow indicates the fragment of interest, whose size is 500 base pairs.

Ninety-seven wood samples from Japanese plum trees showing foliar silvering (Figure 1A) and internal brown circular discolorations in transverse section (Figure 1B) (ragged, tapered cylinders in axial section), in affected branches and main trunks, were collected from 2018 to 2020. Some samples also showed other wood symptoms such as wedge-shaped and dark brown irregular discolorations, suggesting the presence of a diversity of pathogens. In 95 isolates, obtained from 98% of the wood samples, macroscopic and microscopic morphological characteristics of the colonies were consistent with those described for *C. purpureum* [44]. White-cottony mycelia (Figure 1C) with clamp connections grew from wood sections from the necrosis progress area, which were surface disinfected and plated on Petri plates containing acidified potato dextrose agar (APDA) (25% PDA, acidified with 0.2% *v*/*v* 85% lactic acid) and water agar (WA). After 14–21 days of incubation at 25 °C, 52% of the isolates developed beige to light pink pseudo-basidiocarps on the edges of the plates. The fruiting bodies of most of the isolates developed hyaline, apiculate, and ovoid basidiospores, 5.3 (4.7–6.8) µm × 3.6 (3.0–4.5) µm (n = 50).

Moreover, the identity of 57 isolates representing different localities and host cultivars was confirmed though DNA amplification using APN1 primers, developing intensely discrete 500-bp bands on agarose gels (Figure 1D) [14]. The internal transcribed spacer of representative isolates, selected from different host cultivars and collection localities (HMCi7; HMCi121; HMCi147; and HMCi148), was also amplified using Internal Transcribed Spacer sequence (ITS) ITS1 and ITS4 [45]. Sequences were deposited in GenBank (MW938164, MW938165; MW938166; and MW938167, respectively) (Table 1).

In 24% of the wood samples, it was possible to isolate other fungal pathogens, most of them associated with wedge-shaped and irregular dark-brown discolorations. Fungi were identified as *Cytospora* (12.6%), *Phomopsis* (9.2%), *Schizophyllum* (17.2%), and *Stereum* (7.9%) species in the Botryosphaeriaceae family (42.8%), in addition to other fungi (10.3%).

### 2.2. Pathogenicity Tests

**Figure 2:**
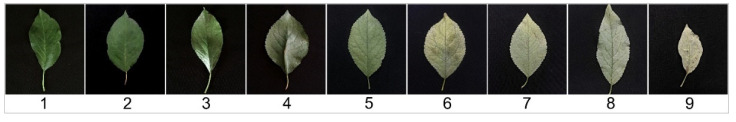
Silverleaf disease foliar symptom visual severity scale (one to nine) for Japanese plum. 1 = healthy or apparently healthy leaf, 3 = epidermis starts detaching from the mesophyll and the color is lighter than in healthy leaves, 5 = more than 75% of the leaf epidermis is detached from the mesophyll and the leaf is beginning to look gray–silver, 7 = 100% of the epidermis is detached from the mesophyll and the leaf is completely white–silver, 9 = the epidermis peels off from the leaf and the mesophyll begins to oxidize. Grades 2, 4, 6, and 8 indicate intermediate symptom severity between the previous and the following grade.

**Figure 3:**
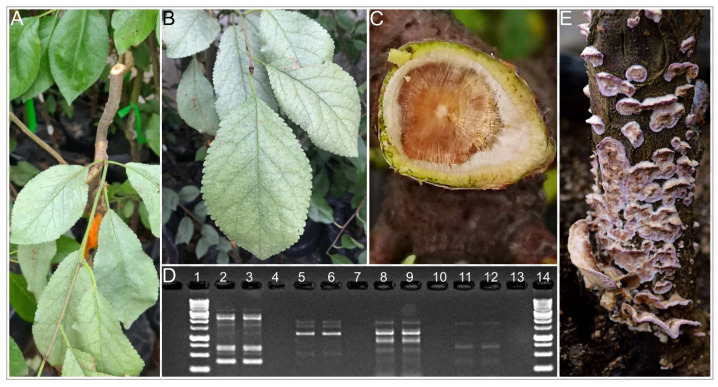
Pathogenicity tests of four *Chondrostereum purpureum* isolates on 2-year old Japanese plum plants ‘Angeleno’; (**A**) transversal cut on an inoculated branch 20 cm from the inoculation point, (**B**) foliar symptoms, and (**C**) necrotic symptoms developed by inoculated plants after a 65-day incubation. (**D**) *Chondrostereum purpureum* DNA banding patterns on agarose gel, after amplification with APM22 fingerprinting markers. Lanes 2 and 3: inoculated and reisolated *C. purpureum* HMCi7, Lanes 5 and 6: isolated and reisolated HMCi121, Lanes 8 and 9: isolated and reisolated HMCi147, and Lanes 11 and 12: isolated and reisolated HMCi148. Lanes 4, 7, 10, and 13 are negative controls. Lanes 1 and 14: molecular weight standards. (**E**) Fruiting bodies of isolate HMCi121 after 20 months of incubation.

Healthy nursery plants were successfully inoculated with *C. purpureum* isolates and reproduced silver foliar symptoms (Grade 3–8) (Figure 2 and Figure 3A,B). After the incubation period, brown central staining was visible when the inoculated branches were transversally cut (Figure 3A,C). Moreover, one of the inoculated plants, with the HMCi121 isolate, developed resupinated purple carpophores in the main trunk, above the soil level, after 20 months of incubation (Figure 3E).

It was possible to reisolate *C. purpureum* from inoculated plants (100%) on APDA, while the fungus was not reisolated from controls. The banding patterns in agarose gels, produced by DNA amplification using Sequence Characterized Amplified Region (SCAR) fingerprinting primers APM22D13 [14], were identical between inoculated and reisolated *C. purpureum* isolates (Figure 3D), fulfilling Koch’s postulates.

### 2.3. Silverleaf Effects on Plum

**Figure 4:**
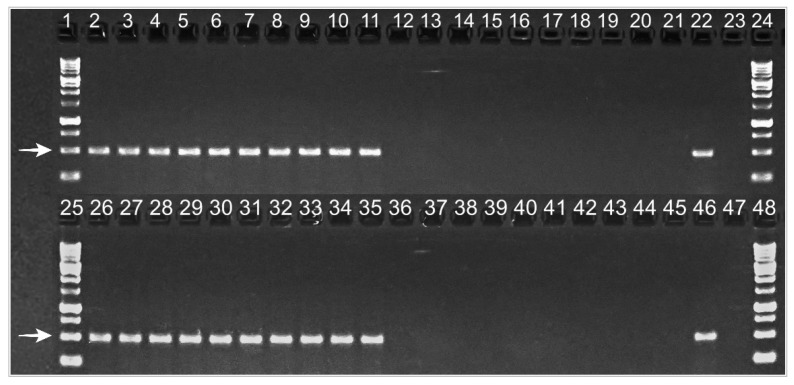
*Chondrostereum purpureum* DNA amplification using APN1 species-specific primers in Silverleaf-diseased plum plants, showing foliar symptoms (Lanes 2–11 and 26–35), and healthy ones (Lanes 12–21 and 36–45) (2019). Lanes 22 and 46: positive controls (isolate HMCi147 GenBank: MW938166). Lanes 23 and 47: negative controls. Lanes 1, 24, 25, and 48: molecular weight standards. White arrows indicate the fragment of interest, whose size is 500 base pairs.

To confirm the presence or absence of *C. purpureum* in symptomatic and asymptomatic plants, respectively, the fungus was detected by amplifying fungal DNA, directly from the trunk sawdust, using APN1 species-specific primers, in 2019 and 2020. A 500-bp band was reproduced in agarose gels from diseased trees, while DNA from asymptomatic ones did not reproduce the band (Figure 4). In 2020, symptoms were less severe than in 2019 (Grade 3, average), and two of the previously selected trees did not show any foliar symptoms, but they were positive for APN1 amplification.

#### 2.3.1. Water Potential

**Figure 5:**
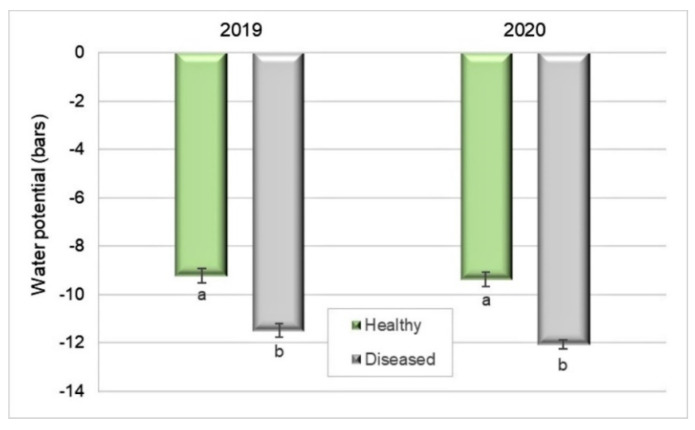
Water potential of leaves of healthy and Silverleaf-diseased Japanese plums ’Angeleno’, based on ten replicates of two trees and five leaf samples per tree (2019) and 9 × 2 × 5 (2020). Bars represent the standard error of the means, and columns with different letters are statistically different. Student’s *t*-test: *p* < 0.0001 in 2019 and 2020.

Xylem potential was 19% lower in diseased plants (−11 bar) than in healthy ones (−9.2 bar) in 2019 (Student’s *t*-test, *p* < 0.0001). Similarly, in 2020, the water potential of diseased plants (−12 bar) was 22.5% lower than that in healthy ones (−9.4 bar) (Student’s *t*-test, *p* < 0.0001) (Figure 5). It was measured a few days after harvest in both years.

#### 2.3.2. Yield Assessment

**Figure 6:**
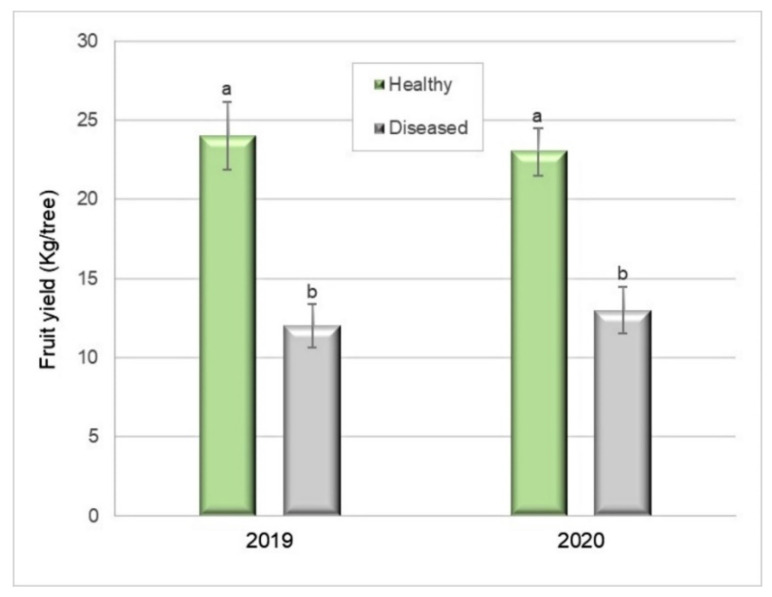
Fruit yield (kg) of healthy and Silverleaf-diseased Japanese plums ’Angeleno’. Ten replicates of two trees (2019) and 9 × 2, in 2020. Bars represent the standard error of the means, and columns with different letters are statistically different. Student’s *t*-test: *p* = 0.0001 in 2019 and *p* = 0.0003 in 2020.

The total yield of the Silverleaf-diseased trees was 51% lower (12.1 kg per tree) than that of healthy ones (24.8 kg per tree) (Student’s *t*-test, *p* = 0.0001) in 2019 (Figure 6). In 2020, the total yield was 41% lower (13.7 kg per tree) than that of healthy plants (23.5 kg per tree) (Student’s *t*-test, *p* = 0.0003) (Figure 6).

#### 2.3.3. Fruit Quality

**Figure 7:**
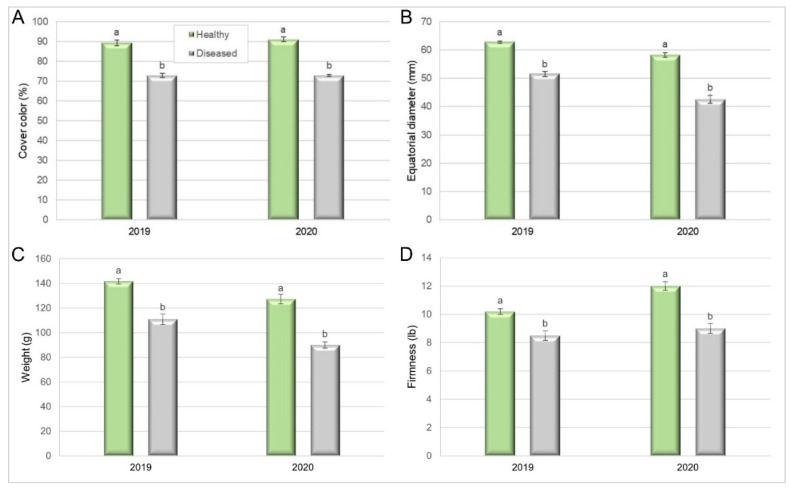
Quality of fruit from healthy and Silverleaf-diseased trees in a Japanese plum orchard (’Angeleno’): (**A**) cover color (Pearson Chi-squared test, *p* = 0.0001 in 2019 and *p* < 0.0001 in 2020), (**B**) equatorial diameter (Kruskal–Wallis, *p* = 0.0002 in 2019; *p* < 0.0001 in 2020), (**C**) individual weight (Student’s *t*-test, *p* < 0.0001 in 2019 and 2020) and (**D**) firmness (Kruskal–Wallis, *p* = 0.0005 in 2019 and *p* < 0.0001 in 2020). Mean of 20 trees × 10 fruit samples (2019) and 18 × 10 (2020). Bars represent the standard error of the means, and columns with different letters are statistically different.

In the 2019 period, fruit harvested from healthy trees had 89% cover colour, compared to 73% in diseased ones (Pearson Chi-squared test = 16.37, *p* = 0.0001). In the next season, the cover colour of fruit from healthy trees was higher than that in the previous year (91%), while the colour of fruit from diseased trees remained similar to that in the previous year (Pearson’s Chi-squared test = 18, *p* < 0.0001) (Figure 7A).

The fruit equatorial diameter was also negatively affected by the disease. In 2019, it was 62.8 mm in fruit from healthy trees, compared to 51.5 mm in fruit from diseased ones (Kruskal–Wallis, *p* = 0.0002). In 2020, the equatorial diameter was lower than in the previous season, both for fruit from healthy trees (58.5 mm) and diseased ones (42.4 mm) (Kruskal–Wallis, *p* < 0.0001) (Figure 7B).

Fruit individual weight was also a parameter affected by Silverleaf. In 2019, fruit harvested from healthy trees weighed 141.7 g compared with 111.3 g for diseased trees (Student’s *t*-test, *p* < 0.0001). In the next season, fruit from healthy trees weighed 127.5 g compared with 90 g for diseased trees (Figure 7C) (Student’s *t*-test, *p* < 0.0001). Finally, fruit from diseased trees (4.54 kgf) was softer than fruit from healthy ones (3.75 kgf), in 2019 (Kruskal–Wallis, *p* = 0.0005), as well as in 2020 (5.44 kgf for fruit from diseased and 3.88 kgf for fruit from healthy trees (Kruskal–Wallis, *p* < 0.0001)) (Figure 7D), indicating a shorter postharvest life for fruit from diseased trees.

The other measured quality parameters (pH, titratable acidity, soluble-solids, and background colour) were not different between fruit harvested from diseased and healthy trees (data not shown).

## 3. Discussion

In the present study, *C. purpureum* was successfully isolated from the necrotic margin of stained wood of Japanese plums trees showing Silverleaf foliar symptoms. The symptoms were similar to those described for other fruit hosts [3,23,46]. *Chondrostereum purpureum* is a primary invader of woody angiosperms and enters its host through a fresh wound, followed by the infection of aggressive saprobic fungi such as *Trametes versicolor* and *Schizophyllum commune* [15,16]. However, *C. purpureum* is still present in these trees and is also able to produce foliar symptoms, as was demonstrated in this study, with the symptom observation and *C. purpureum* molecular detection and isolation.

The isolated fungus developed white-cottony mycelia, and it was also possible to detect it directly in wood, through clear staining bands on agarose gels, when fungal DNA was amplified with APN1 species-specific primers [14]. Moreover, the Japanese plum plants inoculated with *C. purpureum* isolates clearly showed foliar symptoms, developing silver-greyish leaves in the inoculated branches, as well as internal wood necrosis and fruiting bodies. Reisolated fungal specimens showed the same cultural features as the inoculated ones, as well as identical banding patterns on agarose gels when SCAR fingerprinting markers [14] were used to amplify their DNA, thus fulfilling Koch’s postulates.

It was demonstrated that wood diseases alter physiological parameters in different hosts [47,48]. Likewise, in this study, the water potential was lower in diseased plants compared to healthy ones. Similarly, in 2020, the water potential of diseased plants was lower than that in healthy ones. These differences can be explained by *C. purpureum* growth through the xylem tissues of the host [33], as well as from the systematic silvering of leaves [26]. During the infection process, *C. purpureum* produces the occlusion of tree vessels [31]. Moreover, transpiration, stomatal conductance, and leaf area decrease significantly while leaf-silvering intensity increases [26]. The resulting physiological disruption and dehydration, combined with fungal toxins, finally cause the death of the host [21,33,49].

On the other hand, intensity of symptoms may change between seasons, such as in some trees in the present study. Intensity was lower in some plants, despite the presence and viability of the fungus, which was confirmed by PCR and microbiological isolation, as occurred in apple [50], where the reversion of foliar symptoms was first described. Damaged wood on the branches and trunk is the battleground of microorganisms, pathogens, and endophytes. While vascular tissue is being destroyed by pathogens [51], some endophytic microorganisms could be acting as their antagonists and/or inducing plant resistance [50] and thus modulating disease expression [52].

Foliar symptoms remained similar in their intensity (Grades 3–6 on the severity scale) (Figure 2) in most of the diseased analysed trees (95%) during 2019 and 2020. In 2019, a reduction of 51% in total fruit weight was recorded in Silverleaf-diseased plants compared to healthy ones. Moreover, in 2020, the reduction was 41%. The fungal pathogen inhabiting the vessels and necrotic tissue affected the number and weight of fruits per tree. Similarly, fruit yield decreases due to several wood pathogens [41]. The results were consistent with yield assessments performed in other fruit crops in Chile, such as apples and blueberries [4,53]. Although there are authors who point out yield losses due to the damaging effects of *C. purpureum* infections in stone and pome fruits [18,26,36,40,44], as well as blueberries [4], as far as we are concerned, this is the first record about yield losses in Japanese plum related to the detrimental effects caused by *C. purpureum*.

Regarding fruit quality, our results showed that important quality components such as fruit weight, cover colour, equatorial diameter, and firmness were influenced by Silverleaf disease. Similar results were reported by [25,26] in apple orchards, with smaller and lower numbers of fruit, reduced colour, and an increased incidence of physiological damage such as a water core, short post-harvest storage, and softening of fruit. Furthermore, [27] stated that the spread of *C. purpureum* in woody tissues and the loss of photosynthetic capability eventually leads to tree death.

In conclusion, our work has demonstrated that *C. purpureum* alters physiological parameters such as the water potential of Japanese plum trees and negatively impacts fruit yield and quality. The latter can decrease the orchard productivity and, moreover, the fruit harvested from diseased trees does not meet the requirements to be properly commercialized or exported to demanding markets.

## 4. Materials and Methods

### 4.1. Collection of Samples

Collections of wood samples (97) from plum trees showing foliar silvering and internal wood discoloration symptoms were conducted in the Chilean Japanese plum productive area, from the Metropolitana Region (33°42′16.11″ S, 70°59′11.82″ W) to the Ñuble Region (36°37′24.98″ S, 72°0′23.39″ W). Two nurseries and 27 orchards of different Japanese plum cultivars, mainly ‘Angeleno’, ‘Black Amber’, ‘Friar’, ‘Fortune’, and ‘Larry Anne’, were examined. Samples of *Prunus domestica* subsp. *domestica* ‘D’Agen’ and *Prunus domestica* subsp. *italica* ‘Reina Claudia’ were included (Table 1). Symptoms were recorded and photographed.

### 4.2. Isolation and Purification

In the laboratory, bark was removed from the samples, and 0.5 cm wooden pieces were cut from the margin of the discoloration area. These pieces were superficially disinfected using 10% *v*/*v* sodium hypochlorite (4.9% chlorine) for 4 min and aseptically plated on Petri plates containing acidified potato dextrose agar (APDA) (25% potato dextrose agar (PDA), acidified with 0.2% *v*/*v* 85% lactic acid) (PDA Difco, Baltimore, MD, USA) and water agar (WA) (Winkler, Santiago, Chile). Plates were incubated at 25 °C in darkness until mycelial development. Pure cultures were obtained by transferring hyphal tips to fresh PDA plates and incubating them at 25 °C.

### 4.3. Identification and Characterization

The identification was focused on isolates resembling *C. purpureum*. Fungal colonies showing white to beige cottony mycelia were preliminary selected (n = 95). Subsequently, the isolates were identified by their cultural characteristics after 7 and 14 days of incubation on PDA at 25 °C. The morphology of the mycelia, presence of clamp connections, and morphometry of spores from pseudo-basidiocarps were determined using an optical microscope (Eclipse 80i, Nikon, Tokyo, Japan) and the software Nikon NIS-elements D2.30 (Tokyo, Japan).

Furthermore, representative isolates were identified by molecular means (n = 57). Pure cultures were incubated on PDA at 25 °C for 7 days. Total nucleic acids were isolated from fresh mycelium using the CTAB method, and genomic DNA (20 ng) was amplified using APN 1 *C. purpureum*-specific primers (Table 2), following the protocols described by [3]. The PCR products (20 μL) were analysed on 1.5% agarose gels, using a 1 kb DNA ladder (Maestrogen Inc., Xiangshan Dist., Hsinchu, Taiwan) as a molecular size standard. Electrophoresis was performed at 7.5 V/cm for 1 h, and gels were stained with ethidium bromide (1 μg/mL). Gels were visualized under UV light (λ = 365 nm) transillumination (Clear View standard UV transilluminator, UK) and digitally recorded (PC2010, Cannon, Nagasaki, Japan).

The internal transcribed spacer of four representative isolates selected from different host cultivars and collection localities was amplified using ITS1 and ITS4 primers (Table 1) and GoTaq^®^ Green Master Mix 2X (Promega, Madison, WI, USA). The PCR conditions were an initial denaturation at 94 °C for 5 min, followed by 30 cycles of denaturation at 94 °C for 1 min, annealing at 52 °C for 1.5 min, and extension at 72 °C for 2 min. Fungal DNA was quantified using a fluorometer (Qubit 2.0, Invitrogen, Carlsbad, CA, USA). Electrophoresis, staining, and visualization were conducted as described for APN1 gels. The PCR products were purified and sequenced by Macrogen (Macrogen Inc., Seoul, Korea), and the nucleotide sequences were assembled and edited using the Sequencher software version 5.4.6 (Gene Codes Corporation, Ann Arbor, MI, USA). Subsequently, sequences were compared with the GenBank database using the BLAST software (Basic Local Alignment Search Tool program), (National Center for Biotechnology Information (NCBI), Rockville Pike, Bethesda, MD, USA).

Furthermore, colonies showing different characteristics such as a dark colour were preliminary identified by the morphometry of their reproductive structures. Basidiomycete fungi, of which the colonies are similar to those of *C. purpureum*, were discriminated by the colony shape and density and also by their DNA amplification using APN1 species-specific primers [23].

### 4.4. Pathogenicity

Four representative isolates were inoculated on healthy 2-year-old nursery plants ‘Angeleno’. Fresh cuts were aseptically performed on the main 1-year-old lateral branches (1–1.5 cm diameter). Cuts were inoculated with 0.7-cm-diameter mycelial plugs collected from growing colonies of *C. purpureum* representative isolates HMCi7, HMCi121, HMCi147, and HMCi148 and incubated on PDA for 7 days at 25 °C. Sterile agar was used for controls. The inoculum was covered with petroleum jelly and plastic film to prevent dehydration. Five plants were inoculated per treatment based on a completely randomized block design at the beginning of September 2019. Plants were incubated in a screenhouse for 65 days at an environmental temperature of 5–18 °C and were periodically monitored to observe the occurrence of foliar symptoms, of which the severity was recorded using a visual scale (Figure 2). After the incubation period, branches were cut and analysed in the laboratory. Branches were cut longitudinally, and necrotic symptoms were recorded. Small wood pieces 0.5 cm were cut from the necrotic margin and aseptically plated on APDA. The reisolated fungi were purified, following the protocol described above.

Genomic DNA was extracted from the reisolated fungi and amplified using *C. purpureum* SCAR species-specific primers [14] (Table 1). PCR conditions were those indicated by the authors. Electrophoresis, staining, and visualization were conducted as described above. Subsequently, banding patterns from the inoculated isolates were compared with the reisolated ones to confirm Koch’s postulates.

### 4.5. Silverleaf Effects on Plum

To determine the Silverleaf effects on plum trees, water potential, fruit yield, and quality were measured in a 22-year-old orchard (‘Angeleno’) on Mariana 2624 rootstock, with planting distances of 4.5 × 3 m, located in the Maule Region (34°58′58.21″ S, 71°16′37.01″ W), in the 2019 and 2020 harvest periods. Twenty healthy and 20 diseased trees were selected, homogeneous in their height and architecture, and located on four adjacent rows, to avoid topography and other differences among them. Each experimental unit consisted of two adjacent trees of the same treatment, with 10 replicates.

Trees were selected based on the absence of foliar silver symptoms for healthy ones and on the presence of them in diseased ones (Grade 3 or higher on the symptom severity scale) and were widespread throughout the canopy for diseased plants, meaning more than the 80% of the leaves had Silverleaf symptoms, from slightly to severely affected.

The Silverleaf foliar symptom visual severity scale (Grades 1–9) was devised using leaves from ‘Angeleno’ plum trees, naturally infected with *C. purpureum*, showing different levels of Silverleaf symptoms. Grades were assigned to the different foliar symptom visual intensities: 1 = healthy or apparently healthy leaf, 3 = epidermis starts detaching from the mesophyll and the colour is lighter than that in healthy leaves, 5 = more than 75% of the leaf adaxial epidermis is detached from the mesophyll, and the leaf is beginning to look grey–silver, 7 = 100% of the epidermis is detached from the mesophyll, and the leaf is completely white–silver, 9 = the epidermis peels off from the leaf, and the mesophyll begins to oxidize. Grades 2, 4, 6, and 8 indicate intermediate symptom severity between the previous and the following grade. Epidermis detachment on the leaf adaxial surface was confirmed using a Stereo Microscope at 15 × (Olympus SZ61, Tokyo, Japan).

Additionally, the presence or absence of *C. purpureum* was confirmed through the amplification of DNA isolated from sawdust collected from the main trunk [3], using APN1-specific primers [14], following the previously described protocol.

#### 4.5.1. Water Potential

Xylem potential was measured on diseased and healthy trees on 1 March 2019, and 10 March 2020 (about 3–6 days after harvest). For both periods, it was measured on light-exposed leaves (five leaves per plant), which were previously covered for 2 h to avoid sunlight. Xylem potential was measured using a Scholander-type pressure chamber (Model 615, PMS Instruments, Albany, NY, USA).

#### 4.5.2. Yield Assessment

Fruit was harvested on 27 February 2019 and 4 March 2020, the same dates that producers harvested the orchard. Before harvesting the fruit from each selected healthy and diseased tree, 10 random fruits from one lateral representative branch of each tree were set aside for quality parameter analysis (see Section 4.5.3) in the laboratory. Subsequently, the remaining fruit of each tree was individually collected and weighed.

#### 4.5.3. Fruit Quality

Quality parameters were measured on the 10 previously reserved fruit. The measured parameters were individual weight (g), cover colour (%), and background colour (%), based on a plum colour chart [54], calibre (polar and equatorial diameter measured using a digital calliper), titratable acidity measured by potentiometric titration with NaOH 0.1 N (Hanna, pH 211, Nușfalău, Romania) expressed as citric acid proportion (%) (AOAC, 2000), pH, and soluble solids, measured with a digital refractometer (Brix degrees) (Atago, Pocket PAL-refractometer, Japan). For firmness measurement, two cheeks per fruit were cut, and the firmness was measured using a manual firmness penetrometer (FT 327, Facchini, Alfonsine, Italy) inserted in an aluminium holder for penetrometers (Dimeri, Santiago, Chile). The probe diameter was 8 mm. Media from both cheeks were subsequently analysed. The average of measurements (n = 10) was calculated for each parameter for further analysis.

## 5. Experimental Design and Statistical Analyses

The experimental unit consisted of two adjacent trees from the same treatment. The treatments were Silverleaf-diseased and healthy conditions, with 10 replicates from four rows in the orchard based on a randomized complete block design.

Fruit yield (total weight) was directly analysed, while for water potential, 10 samples (leaves) per plot were measured and averaged. Subsequently, averages of each plot were statistically analysed. For each fruit quality parameter, 20 samples (fruits) per plot were measured, and the averages were analysed.

Before testing for statistical significance, to detect differences between treatment means, normality and homoscedasticity were evaluated by the Shapiro–Wilk test, residual independence analysis, and graphical methods. When assumptions of normality and homoscedasticity were fulfilled, Student’s *t*-test was used to compare healthy and diseased means of water potential, yield, and individual fruit weight.

Cover colour was analysed by Pearson’s Chi-squared test, and fruit firmness and calibre by the Kruskal–Wallis test. The statistical analyses used depended on the nature of the data and distribution. Analyses were performed using InfoStat 2021 statistical software (Universidad Nacional de Córdoba, Córdoba, Argentina).

## Figures and Tables

**Table 1:** Isolate, plant host, geographic origin, DNA amplification using APN1 *Chondrostereum purpureum*-specific primers, and GenBank Accession number of 17 representative *C. purpureum* isolates, morphologically and molecularly identified in this study.

Isolate	Species	Host		Geographic Origin		APN1	ITS GenBank Accession Number
HMCi 314	*Chondrostereum purpureum*	*Prunus domestica* subsp. *domestica*	D’Agen	Colbún	35°45′01.1592″ S, 71°25′46.4889″ W	Positive	-
HMCi 325	*Chondrostereum purpureum*	*Prunus domestica* subsp. *domestica*	D’Agen	Sagrada Familia	34°59′50.0820″ S, 71°21′48.2976″ W	Positive	-
HMCi 331	*Chondrostereum purpureum*	*Prunus domestica* subsp. *domestica*	D’Agen	San Javier	35°38′54.7944″ S, 71°36′47.9340″ W	Positive	-
HMCi 341	*Chondrostereum purpureum*	*Prunus domestica* subsp. *italica*	Reina Claudia	Chillán	34°58′10.7536″ S, 71°21′14.8464″ W	Positive	-
HMCi 308	*Chondrostereum purpureum*	*Prunus domestica* subsp. *italica*	Reina Claudia	San Rafael, Maule	35°18′37.0044″ S, 71°29′06.5832″ W	Positive	-
HMCi 290	*Chondrostereum purpureum*	*Prunus domestica* subsp. *italica*	Reina Claudia	Yungay	37°07′21.6127″ S, 72°00′02.1028″ W	Positive	-
HMCi 249	*Chondrostereum purpureum*	*Prunus salicina*	Angeleno	Codegua	34°01′12.3420″ S, 70°41′50.0352″ W	Positive	-
HMCi 7	*Chondrostereum purpureum*	*Prunus salicina*	Angeleno	Curicó	34°58′58.21″ S, 71°16′37.01″ W	Positive	MW938164
HMCi 340	*Chondrostereum purpureum*	*Prunus salicina*	Angeleno	Portezuelo	36°34′43.9356″ S, 72°33′19.7424″ W	Positive	-
HMCi 121	*Chondrostereum purpureum*	*Prunus salicina*	Black amber	Curicó	36°37′27.1128″ S, 72°00′27.8532″ W	Positive	MW938165
HMCi 272	*Chondrostereum purpureum*	*Prunus salicina*	Black amber	Romeral	34°57′17.2836″ S, 71°08′11.2560″ W	Positive	-
HMCi 276	*Chondrostereum purpureum*	*Prunus salicina*	Black amber	Teno	34°52′39.4149″ S, 71°05′19.0032″ W	Positive	-
HMCi 168	*Chondrostereum purpureum*	*Prunus salicina*	Fortune	Melipilla	33°41′09.3696″ S, 71°06′23.7960″ W	Positive	-
HMCi 253	*Chondrostereum purpureum*	*Prunus salicina*	Friar	Paine	33°52′11.7156″ S, 70°44′21.5700″ W	Positive	-
HMCi 148	*Chondrostereum purpureum*	*Prunus salicina*	Larry Ann	Curicó	34°58′56.0352″ S, 71°16′34.0896″ W	Positive	MW938167
HMCi 147	*Chondrostereum purpureum*	*Prunus salicina*	Larry Ann	Rio Claro	35°12′0.18936″ S, 71°14′36.1140″ W	Positive	MW938166
HMCi 157	*Chondrostereum purpureum*	*Prunus salicina*	Larry Ann	Yungay	37°08′50.452″ S, 71°52′20.673″ W	Positive	-

**Table 2:** Primer sequences used in the molecular analysis. APN1 and APM22 are specific primers for *Chondrostereum purpureum*.

Primer	Target	Sense	Sequence (5′-3′)	TM (°C)	Reference
ITS1	ITS	Forward	CTTGGTCATTTAGAGGAAGTAA	51	[45]
ITS4	ITS	Reverse	TCCTCCGCTTATTGATATGC	52	[45]
APN1-F	IGS	Forward	GCACGGAGAAGGAGAAGATTGGCT	61.6	[14]
APN1-R	IGS	Reverse	TTTCGGACTTTTGGGGCTCATTTCG	64.7	[14]
APM22D13F	SCAR	Forward	GGGGTGACGAGGACGACGGTG	63.2	[14]
APM22D13R	SCAR	Reverse	GGGGTGACGACATTATACTGCAGGTAGTAG	60	[14]

## Data Availability

Data is contained within the article.
